# Supplementary material for: A novel and robust feature selection method with FDR control for omics-wide association analysis
Source: PLoS One. 2025 Aug 22;20(8):e0300490. doi: 10.1371/journal.pone.0300490 (PMC12373251; doi:10.1371/journal.pone.0300490)
Supplement: S1 File — (PDF) [file pone.0300490.s001.pdf]

Supplementary Materials for

A novel robust feature selection method with FDR control for omics-wide association analysis

Nov 10, 2023

Correspondence Author: Jian Xiao

1 The proof of Theorem 1

Proof. We mainly borrow the U-statistics tool to implement our proofs.

Firstly, according to the definition of θ^0 (given in the manuscript), we have

$$\theta^0 = \arg\min_{\theta \in R^p} \mathbb{E} Q(\theta). \tag{1}$$

Since the features' covariance matrix Σ_X is positive definite, the minimizer θ^0 is unique and is given by the formula

$$\theta^0 = \frac{1}{n^2} \Sigma_X^{-1} \mathbb{E}[\sum_{i=1}^{n} R_i X_{i,\cdot}]. \tag{2}$$

Now, using the facts that

$$\sum_{i=1}^{n} R_i X_{i,\cdot} = \sum_{i=1}^{n} (\sum_{l=1}^{n} I(Y_l \leq Y_i)) X_{i,\cdot}, \tag{3}$$

then we derive

$$\sum_{i=1}^{n} (\sum_{l=1}^{n} I(Y_l \leq Y_i)) X_{i,\cdot} = \sum_{(i,l):i \neq l} I(Y_l \leq Y_i) X_{i,\cdot} + \sum_{i} X_{i,\cdot} \tag{4}$$

and that $E(X_{i,\cdot}) = 0$, we can write

$$\theta^0 = \frac{n-1}{n} \Sigma_X^{-1} \mu_1, \tag{5}$$

or write θ^* in a asymptotical manner (θ^* is asymptotically equal to θ^0, as $n \to \infty$).

$$\theta^* = \frac{n}{n-1} \theta^0 = \Sigma_X^{-1} \mu_1,$$

where $\mu_1 = \mathbb{E} A_1$ is the expected value of the U-statistic

$$A_1 = \frac{1}{2C_n^2} \sum_{(i,l):i \neq l} I(Y_l \leq Y_i) X_{i,\cdot}. \tag{6}$$

Let $a \doteq a_n$ be a fixed sequence such that $a \to 0$. We can calculate that for every θ

$$Q(\theta^* + a\theta) - Q(\theta^*) = -\frac{a}{n^2} [\sum_{i=1}^{n} R_i X_{i,\cdot}] \theta + a\theta^\top (\frac{X^\top X}{n}) \theta^* + \frac{a^2}{2} \theta^\top (\frac{X^\top X}{n}) \theta, \tag{7}$$

then using (3) with (4), (7) can be written as

$$
\begin{aligned}
&-\frac{a}{n^2}[\sum_{i=1}^{n} R_i X_{i,\cdot}]\theta + a\theta^\top(\frac{X^\top X}{n})\theta^* + \frac{a^2}{2}\theta^\top(\frac{X^\top X}{n})\theta \\
&= -a\frac{(n-1)}{n}A_1\theta - \frac{a}{n^2}\sum_i X_{i,\cdot}\theta + a\theta^\top(\frac{X^\top X}{n})\theta^* + \frac{a^2}{2}\theta^\top(\frac{X^\top X}{n})\theta \qquad (8) \\
&= -a\frac{(n-1)}{n}A_1\theta - \frac{a}{n}\frac{\sum_i X_{i,\cdot}}{n}\theta + a\theta^\top(\frac{X^\top X}{n})\theta^* + \frac{a^2}{2}\theta^\top(\frac{X^\top X}{n})\theta.
\end{aligned}
$$

Because $\mathbb{E}X_{i,\cdot} = \frac{\sum_i X_{i,\cdot}}{n} = \overrightarrow{0}$, thence (8) becomes

$$
\begin{aligned}
&-\frac{a}{n^2}[\sum_{i=1}^{n} R_i X_{i,\cdot}]\theta + a\theta^\top(\frac{X^\top X}{n})\theta^* + \frac{a^2}{2}\theta^\top(\frac{X^\top X}{n})\theta \\
&= -a\frac{(n-1)}{n}A_1\theta + a\theta^\top(\frac{X^\top X}{n})\theta^* + \frac{a^2}{2}\theta^\top(\frac{X^\top X}{n})\theta. \qquad (9)
\end{aligned}
$$

Then we further have

$$
\begin{aligned}
&\frac{1}{a^2}[Q(\theta^* + a\theta) - Q(\theta^*)] = \qquad (10) \\
&= -\frac{1}{a\sqrt{n}}[\frac{(n-1)}{n}\sqrt{n}A_1\theta - \sqrt{n}\theta^\top(\frac{X^\top X}{n})\theta^*] + \frac{1}{2}\theta^\top(\frac{X^\top X}{n})\theta \\
&= -\frac{1}{a\sqrt{n}}[\frac{(n-1)}{n}[\sqrt{n}(A_1\theta - \mu_1\theta)] - \sqrt{n}[\theta^\top(\frac{X^\top X}{n})\theta^* - \mu_1\theta]] + \frac{1}{2}\theta^\top(\frac{X^\top X}{n})\theta.
\end{aligned}
$$

Then using LLN, Lemma 1 (given below) and Slutsky's theorem we get that

$$
\frac{1}{a^2}[Q(\theta^* + a\theta) - Q(\theta^*)] \to_{f-d} \frac{1}{2}\theta^\top\Sigma_X\theta + b\theta^\top\mathbb{W}, \qquad (11)
$$

where $\to_{f-d}$ denotes the finite-dimensional convergence in distribution, and $\mathbb{W} \sim N(0, D)$ with D defined in the following Lemma 1.

Then for (11), let $a_n = \frac{1}{\sqrt{n}}$, we have $b = 1$ and

$$
n[Q(\theta^* + a\theta) - Q(\theta^*)] \to_{f-d} \frac{1}{2}\theta^\top\Sigma_X\theta + \theta^\top\mathbb{W}. \qquad (12)
$$

Furthermore, by minimizing the above (12) and the idea (Geyer, 1996), we can have

$$
\sqrt{n}(\widehat{\theta}_{\mathrm{ROLS}} - \frac{n}{n-1}\theta^0) = \sqrt{n}(\widehat{\theta}_{\mathrm{ROLS}} - \theta^*) \qquad (13)
$$

$$\to_d \arg\min_\theta (\frac{1}{2}\theta^\top \Sigma_X \theta + \theta^\top \mathbb{W}).$$

Then by (13), we can get

$$\sqrt{n}(\widehat{\theta}_{\text{ROLS}} - \frac{n}{n-1}\theta^0) \to_d -\Sigma_X^{-1}\mathbb{W}. \tag{14}$$

Furthermore, we have

$$\frac{(\widehat{\theta}_{\text{ROLS},j} - \frac{n}{n-1}\theta_j^0)}{\sqrt{\text{Var}(\widehat{\theta}_{\text{ROLS},j})}} \to_d N(0,1), \tag{15}$$

where $\text{Var}(\widehat{\theta}_{\text{ROLS},j}) = [\Sigma_X^{-1} D \Sigma_X^{-1}]_{j,j}$ and $[\Sigma_X^{-1} D \Sigma_X^{-1}]_{j,j}$ denotes the j-th diagonal element in the matrix $[\Sigma_X^{-1} D \Sigma_X^{-1}]$, and D is defined by the Lemma 1 in the supplementary materials.

Note that $\mathbb{E}(\widehat{\theta}_{\text{ROLS},j}) = \frac{n}{n-1}\theta_j^0 \to \theta_j^0$, thence $\widehat{\theta}_{\text{ROLS},j}$ is the asymptotically unbiased estimates of θ_j^0. Thus, under null hypothesis: $\theta_j^0 = 0$, the estimator $\widehat{\theta}_{\text{ROLS},j}$ follows asymptotically normal distribution with symmetry around 0 property under null hypothesis. Furthermore, the estimators $\widehat{\theta}_{\text{ROLS},1}$ and $\widehat{\theta}_{\text{ROLS},2}$ obtained by the two different part samples respectively ensure the statistics $T_{1,j}$ and $T_{2,j}$ are asymptotically normal with symmetry around zero under the null hypothesis. Then the symmetry around zero property of statistics $W_j = T_{1,j}T_{2,j}$ is ensured because of the fact that $T_{1,j}$ is independent of $T_{2,j}$.

2 Lemma 1 and the proof of Lemma 1

Lemma 1. Suppose that Assumptions 1, 2, 3 and 4 of the manuscript are satisfied, $\mathbb{E}|X_{i,\cdot}|^4 < \infty$ and Σ_X is positive definite, then

$$[\sqrt{n}(A_1\theta - \mu_1\theta)] - \sqrt{n}[\theta^\top (\frac{X^\top X}{n})\theta^* - \mu_1\theta] \to_d \mathbb{W}$$

and

$$\mathbb{W} \sim N(0, D),$$

where D are stated precisely in the proof below.

Proof. Consider U-statistics A_1 defined in the above section, and $(\frac{X^\top X}{n})\theta^*$ also can be viewed as a U-statistics. Especially,

$$\mathbb{E}[(\frac{X^\top X}{n})\theta^*] = \Sigma_X \mathbb{E}[\theta^*] = \mu_1.$$

Define the kernel function f_1 and f_2 for the U-statistics A_1 and $(\frac{X^\top X}{n})\theta^*$ as

$$f_1 = \frac{1}{2} \sum_{(i,l),i \neq l} I(Y_l \leq Y_i) X_{i,\cdot},$$

$$f_2 = \sum_{i=1}^{n} \frac{X_{i,\cdot}^\top X_{i,\cdot}}{n} \theta^*.$$

Using Theorem 7.1 (hoeff, 1948) we obtain convergence in distribution in R^{2p}

$$\sqrt{n} \begin{bmatrix} A_1 - \mu_1 \\ (\frac{X^\top X}{n})\theta^* - \mu_1 \end{bmatrix} \sim N(0, \Sigma)$$

for the matrix

$$\Sigma = \begin{pmatrix} \overbrace{\Sigma_{11}}^{p \times p} & \overbrace{\Sigma_{12}}^{p \times p} \\ \Sigma_{21} & \Sigma_{22} \end{pmatrix},$$

where for $j, k = 1, \ldots, 2$, Σ_{jk} denotes the covariance matrix between j-th U-statistics and k-th U-statistics, and Σ_{dd} denotes the covariance matrix of d-th U-statistics. The detailed forms of Σ_{jk} and Σ_{dd} can be derived based on the above defined kernel function f_d by following the works (Rejchel and Bogdan, 2020). Because in this paper we use the bootstrap sampling method to estimate the variances of the estimators of the coefficients β, we do not need to derive the detailed forms of Σ.

Next, define $(p \times 2p)$-dimensional matrix M in the following way: for $j = 1, \ldots, p$ put $M_{j,j} = 1$ and for $i = 1, \ldots, p$, $M_{i,p+i} = -1$, and zeros elsewhere. Then

$$[\sqrt{n}(A_1 - \mu_1)] - \sqrt{n}[(\frac{X^\top X}{n})\theta^* - \mu_1] \rightarrow_d \mathbb{W}$$

with

$$\mathbb{W} \sim N(0, M\Sigma M^\top),$$

and

$$\Sigma = \begin{pmatrix} \overbrace{\Sigma_{11}}^{p\times p} & \overbrace{\Sigma_{12}}^{p\times p} \\ \Sigma_{21} & \Sigma_{22} \end{pmatrix}.$$

Then let $D = M\Sigma M^\top$. □

3 The proof of Theorem 2

Proof. For the rank-based approach single index model with noise variables having any unknown distribution, Theorem 2 (Rejchel and Bogdan, 2020) provides the estimation accuracy result under CIF condition. Then we combine the estimation accuracy result with beta-min assumption $\min_{j\in S}|\theta_j^0| > \frac{4\xi\lambda}{\xi+1}$ to obtain screening property.

Under Assumptions 1, 2, 3 and 4, let $a \in (0,1)$ be arbitrary and $q \geq 1$. Theorem 2 (Rejchel and Bogdan, 2020) shows that there exists a universal constant $K_3 > 0$ such that with probability at least $1 - K_3 a$ we have

$$||\widehat{\theta}_{\text{RLasso}} - \theta^0||_q \leq \frac{4\xi p_0^{1/q}\lambda}{\xi+1}. \tag{16}$$

Let $q = \infty$ for (16), we have

$$||\widehat{\theta}_{\text{RLasso}} - \theta^0||_\infty \leq \frac{4\xi\lambda}{\xi+1}. \tag{17}$$

Because beta-min assumption $\min_{j\in S}|\theta_j^0| > \frac{4\xi\lambda}{\xi+1}$ holds, we have $\lim_{n\to\infty} P(S \subset \widehat{S}) \to 1$, where $\widehat{S} \doteq \{i, \widehat{\theta}_{\text{RLasso},j} \neq 0, i = 1, \ldots, p\}$, and $a \doteq a_n$ is a fixed sequence such that $a \to 0$. This conclusion can be proved by the following procedure.

Suppose that there is a $j \in S$ with $j \notin \widehat{S}$. Then $|\widehat{\theta}_{\text{RLasso},j} - \theta_j^0| = |\widehat{\theta}_{\text{RLasso},j}| > \frac{4\xi\lambda}{\xi+1}$, using the beta-min assumption. On the other hand,

$$|\widehat{\theta}_{\text{RLasso}} - \theta_j^0| \leq ||\widehat{\theta}_{\text{RLasso}} - \theta_j^0||_\infty \leq \frac{4\xi\lambda}{(\xi+1)}$$

which leads to a contradiction. Thence we can obtain the sure screening property

$$\lim_{n\to\infty} P(S \subset \widehat{S}) \to 1.$$

4 The proof of theorem 3

Proof. Because Theorem 1 shows the SDE-ROLS estimate results by the second part samples ensure the statistics $T_{1,j}$ and $T_{2,j}$ are asymptotically normal with symmetry around 0 under the null, the symmetric about zero property of statistics W_j is ensured. Then this symmetric property makes tight control of Δ_js and leads to effective FDR control. Then we can follow proofs of their theorem 4.1 (Du et al., 2021) to prove our Theorem 3 easily. Here we omit this proof procedure.

References

[Du et al., 2021] Du, L. L., Guo, X., Sun, W. G., and Zou, C. L. (2021). False discovery rate control under general dependence by symmetrized data aggregation. *Journal of the American Statistical Association* published online: https://doi.org/10.1080/01621459.2021.1945459.

[Bai, 2003] Bai, J. S. (2003) Inferential theory for factor models of large dimensions. *Econometrica*, **71**(1):135–171, 2003.

[Geyer, 1994] Geyer, C. J. (1994) On the asymptotics of constrained M-estimation. *Annals of Statistics*, **22**:1993–2010, 1994.

[Rejchel and Bogdan, 2020] Rejchel, W. and Bogdan, M. L. (2020) Rank-based lasso efficient methods for high-dimensional robust model selection. *J Mach Learn Res* **21**: 1-47 http://jmlr.org/papers/v21/20-120.html.

[Hoeff, 1948] Hoeffding, W. (1948) A class of statistics with asymptotically normal distribution. *Annals of Mathematical Statistics*, **19**: 293-325, 1948.

[Geer, 2008] Geer, S. vande, (2008) High-dimensional generalized linear models and the lasso. *Annals of Statistics* **36**: 614-645.

[Buhlmann and Mandozz, 2014] Buhlmann, P. Mandozz, J. (2014) High-dimensional variable screening and bias in subsequent inference, with an empirical comparison. *Comput Stat* **29**:407C430 DOI 10.1007/s00180-013-0436-3

[Geer, 2016] Van de Geer, S. *Estimation and Testing under Sparsity.* Springer, 2016.
