# Supplementary material for: A novel and robust feature selection method with FDR control for omics-wide association analysis
Source: PLoS One. 2025 Aug 22;20(8):e0300490. doi: 10.1371/journal.pone.0300490 (PMC12373251; doi:10.1371/journal.pone.0300490)
Supplement: Attachment — Submitted filename: Reviewer comment_R1.pdf [file pone.0300490.s006.pdf]

Dear Editor and Authors,

Thank you for letting me review the revised version of the manuscript. It was very pleasant to see that the authors addressed most of the issues raised in the first review. Now the contents on the paper are clearer and easier to understand. Also, I appreciate that the authors also added the section that provides the theoretical discussion of their methods along with proofs in the supporting information.

Although the manuscript looks good in general, I have some comments on the notations and writing in the manuscript. Please see below:

Line 146: “distributional” → “distribution of”.

Line 179: “I” → “we”.

Line 185: “gotten” → “obtained”.

Line 191: “usually do not satisfy asymptotically symmetric around zero property” → “are usually not asymptotically symmetric around zero”.

Line 224 and 228: I would suggest the authors distinguish the notations for the candidate features for samples ζ_1 and ζ_2. For example, instead of using X^{S_0}, the authors can consider $X_{\zeta_1}^{S_0}$ and $X_{\zeta_2}^{S_0}$.

Line 237 - 239 "It is well known -- complex distribution variable": I would suggest adding references that discuss that bootstrap re-sampling can be used to obtain consistent estimate of the variance.

Line 299: “Theory properties” → “Theoretical properties”.

Line 302: Is $X_{i,\cdot}$ a vector in $\mathbb{R}^p$? Please specify. If so, the authors would need to use the transpose of $X_{i,\cdot}$, i.e., $X_{i,\cdot}^{\mathrm{T}}\beta$ instead of $X_{i,\cdot}\beta$.

Line 312-313: $X_{i,\cdot}^{S}$ and $X_{i\cdot}$ would merely be observations, not "sets". Please use an alternative notation to denote sets.

Line 305 - 306: For real numbers, I suggest using $\mathbb{R}$. Also, please make the notations consistent. On line 306, there are two expressions for expectation: E and $\mathbb{E}$.

Also, is d_θ a real number or a function whose range is in $\mathbb{R}$?

Line 356: “non matter” → “no matter”.
