# Supplementary material for: A novel and robust feature selection method with FDR control for omics-wide association analysis
Source: PLoS One. 2025 Aug 22;20(8):e0300490. doi: 10.1371/journal.pone.0300490 (PMC12373251; doi:10.1371/journal.pone.0300490)
Supplement: Attachment — Submitted filename: Response to Reviewers.pdf [file pone.0300490.s007.pdf]

Response to the comments of reviewer

Thank you very much for your helpful and constructive comments on our manuscript. We have amended our manuscript following your valuable suggestions. Specific replies to your comments are given below:

Overall, the manuscript now has enriched content and appears to suffice the standards required for publication in PLOS One. I recommend that the paper be accepted for publication.

Abstract :

Line 4: "associated relationships" → "association"

Line 10: "can be connected" → "to be connected"

Response: Thanks very much! We have revised all the above problems in the new manuscript in red.

The main body of the manuscript:

Line 11: (Erase "is") omics features is much larger → omics features much larger

Line 11: (Add "which") also poses new → which also poses new

Line 17: (Erase "done by") can be done by based on → can be based on

Response: Thanks very much! We have revised all the above problems in the new manuscript in red.

Line 95: (Recommend using an alternative term for "notable", e.g., useful) hence it enjoys many notable theoretical properties. Thence it enjoys many useful theoretical properties

Response: Thanks very much! We have revised this problem in the new manuscript in red.

Line 101: to valid → to validate
Line 131: Add citation number for "Rejchel et al."

Response: Thanks very much! Sorry for my careless! We have revised this problem in the new manuscript in red.

Line 133: η_{i} is not used in any expression above.

Response: Thanks very much! Sorry for my careless! We have deleted it.

Line 137: The subscript in the definition for θ_0 should be R^P instead of RP.

Line 304: For consistency, replace E() by **E**().

Response: Thanks very much! We have revised this problem in the new manuscript in red.

Line 342: asymptotically symmetry $\longrightarrow$ asymptotically symmetric

Line 344: In Theorem 1, considering that $X_{i,\bullet} = (X_{i,1}, \ldots, X_{i,\mathrm{p}})^{\mathrm{T}}$ is a vector, $E\,|\,X_{i,\bullet}\,|^4 < \infty$ should be revised. For example, it could be $E\,|\,X_{i,\mathrm{j}}\,|^4 < \infty, \mathrm{j} = 1, \ldots, \mathrm{p}$.

Response: Thanks very much! We have revised this problem in the new manuscript in red.

Line 395, 401, and 559: "following the work[]" can be elaborated with author names, for example, "following the work of Rejchel et al. [32]".

Line 556:"then" $\longrightarrow$"thus"?

Response: Thanks very much! We have revised this problem in the new manuscript in red
