# Supplementary material for: A novel and robust feature selection method with FDR control for omics-wide association analysis
Source: PLoS One. 2025 Aug 22;20(8):e0300490. doi: 10.1371/journal.pone.0300490 (PMC12373251; doi:10.1371/journal.pone.0300490)
Supplement: Attachment — Submitted filename: Review_Comment2.pdf [file pone.0300490.s008.pdf]

Dear Editor,

Thank you for the opportunity to review the revised manuscript on the robust feature selection method with FDR control for omics-wide association analysis.

I appreciate the efforts put in by the authors for revising the manuscript based on the reviewer's comments. The contents are much clearer, and the mathematical expressions are easier to follow.

Upon reading the paper line by line, I have only had some minor comments. These are not necessarily crucial in following the writing, but still stand out and could be disturbing for future readers to some extent. Please find the line-by-line comments below. In particular, the comment regarding Line 133 should be addressed.

Overall, the manuscript now has enriched content and appears to suffice the standards required for publication in PLOS One. I recommend that the paper be accepted for publication.

Abstract:

Line 4: "associated relationships" → "association"

Line 10: "can be connected" → "to be connected"

The main body of the manuscript:

Line 11: (Erase "is") omics features is much larger → omics features much larger

Line 11: (Add "which") also poses new → which also poses new

Line 17: (Erase "done by") can be done by based on → can be based on

Line 95: (Recommend using an alternative term for "notable", e.g., useful) hence it enjoys many notable theoretical properties → hence it enjoys many useful theoretical properties

Line 101: to valid → to validate

Line 131: Add citation number for "Rejchel et al."

Line 133: η_i is not used in any expression above.

Line 137: The subscript in the definition for θ_0 should be $\mathbb{R}^p$ instead of R^p.

Line 304: For consistency, replace E() by $\mathbb{E}$().

Line 342: asymptotically symmetry → asymptotically symmetric

Line 344: In Theorem 1, considering that $X_{i,\cdot} = (X_{i,1}, X_{i,2}, \cdots, X_{i,p})^T$ is a vector, $\mathbb{E}|X_{i,\cdot}|^4 < \infty$ should be revised. For example, it could be $\mathbb{E}|X_{i,j}|^4 < \infty$ for $j = (1,2,3, \cdots, p)$.

Line 395, 401, and 559: "following the work [##]" can be elaborated with author names, for example, "following the work of Rejchel et al. [32]".

Line 556: "then" → "thus"?
