# Supplementary material for: A novel and robust feature selection method with FDR control for omics-wide association analysis
Source: PLoS One. 2025 Aug 22;20(8):e0300490. doi: 10.1371/journal.pone.0300490 (PMC12373251; doi:10.1371/journal.pone.0300490)
Supplement: Attachment — Submitted filename: Response to Reviewers.pdf [file pone.0300490.s009.pdf]

Response to the comments of reviewer

Thank you very much for your helpful and constructive comments on our manuscript. We have amended our manuscript following your valuable suggestions. Specific replies to your comments are given below:

Line146:"distributional" $\rightarrow$ "distribution of".

Line 179: "I" $\rightarrow$ "we".

Line185: "gotten" $\rightarrow$ "obtained".

Line 191: "usually do not satisfy asymptotically symmetric around zero property" $\rightarrow$ "are usually not asymptotically symmetric around zero".

Line 224 and 228: I would suggest the authors distinguish the notations for the candidate features for samples ς_1 and ς_2. For example, instead of using X^{S_0}, the authors can consider $X_{\varsigma_1}^{S_0}$ and $X_{\varsigma_2}^{S_0}$.

Response: Thanks very much! We have revised all the above problems in the new manuscript in red.

Line 237 - 239 "It is well known -- complex distribution variable": I would suggest adding references that discuss that bootstrap re-sampling can be used to obtain consistent estimate of the variance.

Response: Thanks very much! We have added the following previous references in the new manuscript in red.

1、DebashisKushary. Bootstrap Methods and Their Application. Technometrics, 2000, 42(2):216-217.
2、Efron B. Bootstrap Methods: Another Look at the Jackknife, Annals of Statistics, 1979, 7(1):1-26.
3、Johnson R W. An Introduction to the Bootstrap. Teaching Statistics, 2010, 23(2):49-54

Line 299: "Theory properties" $\rightarrow$ "Theoretical properties".

Line 302: Is $X_{i,\cdot}$ be a vector in R^p. Please specify. If so, the authors would need to use the transpose of $X_{i,\cdot}$, i.e., $X_{i,\cdot}^T\beta$ instead of $X_{i,\cdot}\beta$.

Response: Thanks very much! We have revised all the above problems in the new manuscript in red.

Line 312-313: $X_{i,\cdot}^S$ and $X_{i,\cdot}$ would merely be observations, not "sets". Please use an alternative notation to denote sets.

Response: Thanks very much! We have revised in the new manuscript in red.

Line 305 - 306: For real numbers, I suggest using R. Also, please make the notations consistent. On line 306, there are two expressions for expectation: E and E.

Also, is d_θ a real number or a function whose range is in R ?

Line 356: "non matter" $\rightarrow$ "no matter".

Response: Thanks very much! We have revised all the above problems in the new manuscript in red.
