# Supplementary material for: A novel and robust feature selection method with FDR control for omics-wide association analysis
Source: PLoS One. 2025 Aug 22;20(8):e0300490. doi: 10.1371/journal.pone.0300490 (PMC12373251; doi:10.1371/journal.pone.0300490)
Supplement: Attachment — Submitted filename: Response_to__reviewers_auresp_4.pdf [file pone.0300490.s010.pdf]

Response to the comments of reviewer

Thank you very much for your helpful and constructive comments on our manuscript. We have amended our manuscript following your valuable suggestions. Specific replies to your comments are given below:

Reviewer #1: Thank you for the opportunity to read and review your work. I carefully read the revised manuscript and I do not have any further comments.

Reviewer #3:

1. Introduction part: the authors utilize rank-based approach and SDA approach to develop a robust feature selection method for a single index model (SIM). Meanwhile, the authors introduce several methods of FDR controlled feature selection: Knockoff filter-based approach, Symmetrized data aggregation (SDA) approach, Stability selection approach. Based on these conceptions, can authors provide rationelle for integrate rank/SDA methods into SIM in the introduction part?

Response: Thanks very much! We have provided some discussions in the revised manuscript in red between the lines 94 and 119.

2. Results part: as observed from results, SIM-FDR is not always ranked to be highest in both aspects of FDR as well as Power. Can authors use a kind of overall/balanced evaluation method to judge that SIM-FDR is best when compared to other methods?

Response: Thanks very much!

1. We first introduced the new measurement: Matthews correlation coefficient (MCC) to evaluate the performance of feature selection. MCC measures the overall accuracy of selection for true positives (TP), false negatives (FN), true negatives (TN), and false positives (FP), with a larger value indicating overall better selection (Baldi et al. 2000; Chicco and Jurman 2020). We have added some descriptions about MCC between the lines 476 and 477 in the revised manuscript in red.
2. The simulation results evaluated via the Matthews correlation coefficient (MCC) are shown in the revised manuscript in red between the lines 561 and 576.

3. Results part: since massive human genomic data sets makes the dimensionality of omics features much larger than its sample size, can authors also use real human genomic data sets to test performance of SIM-FDR?

Response: Thanks very much! We utilized the real Head and neck squamous cell carcinoma (HNSCC) data to further evaluate the performance of the proposed method. The Cancer Genome Atlas profiled 279 head and neck squamous cell carcinomas (HNSCC) to provide a comprehensive landscape of somatic genomic alterations. We have added the analysis of HNSCC data into the revised manuscript in red between the lines 641 and 695.

4. Discussion: list ponits as above

Response: Thanks very much! We have reconstructed the “Discussion” section in the revised manuscript in red.between the lines 696 and 739.

.
