# Supplementary material for: A novel and robust feature selection method with FDR control for omics-wide association analysis
Source: PLoS One. 2025 Aug 22;20(8):e0300490. doi: 10.1371/journal.pone.0300490 (PMC12373251; doi:10.1371/journal.pone.0300490)
Supplement: Attachment — Submitted filename: Reviewer comment.pdf [file pone.0300490.s005.pdf]

Dear Editor,

I would like to thank the authors and the reviewer for letting me review this paper that proposes a very interesting and useful feature selection method that relies on few assumptions. The authors suggest a version of SDA (Du et al. 2023) that uses RankLasso (Rejchel et al. 2020) and rank OLS based on the single index model. The proposed method relies on minimal assumptions, and from the simulation study, this controls for the FDR and at the same time has high power. However, there are some comments that can be addressed to minimize the confusion about the proposed method and could potentially enhance the quality of the contents in the paper. Please find the comments below.

**Comments:**

1. Authors use "model-free" to characterize their proposed approach. The authors in particular refer to model 1 as "model-free". However, despite that there are less restrictions such as linearity in association between Y and X or distribution of the error term, there are model specifications such as monotonicity of g() and that the g() is a function of the linear combination of covariates. Also, the original inventor of this method Rejchel et al. (2020) refer to this model as "robust" rather than "model-free". The SDA (Du et al. 2023) is implemented which may endow a model-free framework in the end. But, this is done after the parameters are estimated from RankLasso and rank OLS based on the single-index model. The authors may consider other expressions to characterize the method or model 1 such as "robust", "distribution-free", etc.

2. Please state the assumptions. Despite there are less model assumptions in the authors' proposed method, there would be assumptions such as iid of samples or the independence between the predictors and the error term. The authors may also consider describing some of the regularity conditions that relate to the SDA or

3. The authors provide knockoff filter-based approach and SDA as two methods that control FDR for feature selection in high-dimensional setting. The stability selection can also be introduced as part of the literature review since it is also a distribution-free feature selection method that efficiently controls for the FDR. Meinhausen and Buhlmann (2010) provides an extensive discussion on this.

4. The authors could discuss some of the limitations of their approach. For example, their method

does not address estimation of the link function and the coefficients (β) for predicting the response. Also, relying on the monotonicity of the link function could also be a limitation.

5. The terms such as "model 1", "problem 2", "problem 3" etc. could be (1), (2), (3), etc., or Model (1), Problem (2), Problem (3), etc.

6. In the simulation study, the authors only consider scenarios in which the single-index is the ground truth, i.e., the linear combination of the covariates are associated with the response. There could be situations in which a double-index or multi-index model is needed. Also, the true link function may not be monotonic in real world. Exploring or deliberating upon the resilience of the single-index model in such circumstances would prove valuable.

7. RankLasso already performs feature selection. This also shows decent performance in controlling for FDR in their simulation study in Rejchel et al. (2020). The authors extend this method by combining with SDA (Du et al.) with multiple sample splitting iterations to control for the FDR. The authors need to clearly specify, how this extension of RankLasso is meaningful by adding more sentences or running simulations.

8. Lines 19 – 23: The sentence that starts by "It generalizes" and ends with "unknown distribution" is very hard to follow. The authors can either break this into two or more sentences or make it concise.

9. Line 31: "makes such approach has lower power" would be "makes such approach to have lower power".

10. Line 49: The knock-off filter would assume that the joint distribution of design matrix X is known. It does not necessarily assume that it is multivariate Gaussian.

11. Line 71: What would be some specific "inappropriate results"? For example, does this indicate wrong feature selection?

12. Lines 73-76: The sentence is too long and hard to follow.

13. Lines 82-87: This long sentence needs to be proof-read. For example, "in the presence of the sample size is moderate" can be revised with more appropriate English phrase.

14. Line 105-106: The authors state that there is no assumption on the link function g(). But, there is the monotonicity assumption.

15. Lines 110-111: The expression for the penalty term in (2) should have double bars -- $|| \, ||_1$.

16. Line 140: The authors refer to the case of $p < n$ as a small number of features. However, the number of features may be close to n or not sufficiently small for a classic least squares methods

could work. In this case, they might also have to use LASSO, or any other high-dimensional method.

17. Line 142: Does "2" refer to "problem 2"?

18. Line 150: It looks like the authors are estimating $\hat{\theta}_{ROLS}$ to apply a threshold from which feature selection is performed. Does this same procedure apply to $\hat{\theta}_{RLasso}$? It would make the idea clearer to readers if the authors provide some more details. Also, the authors propose utilizing stepwise regression to eliminate features that may result in multicollinearity. What is the rationale that using stepwise regression would resolve the multicollinearity issue? Have the authors considered other potential approaches such as evaluating variation inflation factor? What if a covariate should be incorporated in the model despite multicollinearity since, for example, it is a confounder? Especially when the feature size (p) is moderate relative to the sample size, the stepwise feature selection may not perform well. Also, the stepwise method is often performed for feature selection rather than to address multicollinearity, and the method itself accompanies multiple testing issue. Perhaps, the authors can consider proposing the weighted Lasso as proposed in Rejchel et al. (2020) to address low-dimensional setting.

19. Line 155: First, Theorem 11 of Rejchel et al. (2020) has assumptions. Please provide these assumptions. Moreover, the mean of an asymptotic normal distribution would not have the sample size term $n$ in its expression. The expression provided in Appendix A of Rejchel et al. (2020) is not the asymptotic quantity.

20. Line 185: It would be "$2^{nd}$", not "2th".
